# Canine visceral leishmaniasis in the Northeast Region of Brazil

**DOI:** 10.1186/s40409-016-0069-4

**Published:** 2016-04-12

**Authors:** Flávio Gonçalves Brito, Helio Langoni, Rodrigo Costa da Silva, Tereza Emmanuelle de Farias Rotondano, Marcia Almeida de Melo, Giselle Souza da Paz

**Affiliations:** São Paulo State University (UNESP – Univ Estadual Paulista), Botucatu, São Paulo Brazil; Mississippi State University of Agriculture and Applied Science, Starkville, MS USA; Federal University of Pernambuco, Pernambuco, Brazil; Federal University of Campina Grande, Campina Grande, Paraíba Brazil; Diagnostic Service in Zoonosis, Department of Veterinary Hygiene and Public Health, School of Veterinary Medicine and Animal Husbandry, São Paulo State University (UNESP – Univ Estadual Paulista), District of Rubião Junior, s/n, CEP 18.618-970, Botucatu, São Paulo Brazil

**Keywords:** Kala-azar, Diagnosis, Dog, Zoonosis, Risk factors

## Abstract

**Background:**

Visceral leishmaniasis (VL) is a zoonosis that affects dogs and other mammals, including humans. Contact with dogs is a major risk factor for humans. This disease is endemic in several regions of Brazil. The aim of this study was to determine the prevalence of *Leishmania* spp. infection in dogs and to correlate it with possible risk factors.

**Methods:**

Blood samples were collected from 391 dogs of different ages, breeds, and both genders, coming from Campina Grande, Paraíba state, Brazil. An epidemiological questionnaire was employed in order to identify risk factors associated with the disease. Serological tests were performed using indirect immunofluorescence, enzyme-linked immunosorbent assay (ELISA S7®) and polymerase chain reaction.

**Results:**

*Leishmania* spp. antibodies were detected in 33 (8.4 %) and 17 (4.3 %) dogs according to the indirect immunofluorescence test (IFAT) and enzyme-linked immunosorbent assay (ELISA S7®), respectively. PCR results indicated the presence of *L. chagasi* DNA in only eight (2 %) blood samples. There was a significant association between reactive animals and contact with animals from different houses (OR = 4.1; *p* = 0.02).

**Conclusions:**

It is suggested that CVL may occur in urban areas. Moreover, it is demonstrated that the association among different diagnostic tests may lead to a more accurate identification of positive animals, which might help to improve the disease control and prevent euthanasia in false-positive results.

## Background

Leishmaniasis is a worldwide zoonotic disease, transmitted by Phlebotominae sand flies of the Psychodidae family. It is more common in tropical and subtropical areas since its vectors are widely distributed among hot and moderate climate regions [1]. This disease is caused by a dimorphic protozoan belonging to the genus *Leishmania*. In Brazil, visceral leishmaniasis (VL) is caused by *Leishmania (Leishmania) chagasi* and shows a wide territorial distribution among areas of different geographic, climatic and social aspects [2]. Dogs are considered important reservoirs in endemic areas [3]. The prevalence of canine visceral leishmaniasis (CVL) in the Brazilian Northeast region, ranging from in 16 % in Garanhuns to 40.3 % in Paulista, Pernambuco state [4, 5].

There are a few studies related to leishmaniasis prevalence in the city of Campina Grande and epidemiological surveys are the basis for the disease control programs. Thus, this study aimed to investigate the infection with *L. chagasi* in dogs from the urban area of Campina Grande, Paraíba State, and the interpretation of the results by the evolution of an epidemiological survey.

## Methods

Blood samples were collected from 391 dogs, regardless of gender, breed and age, all from private properties from the urban area of Campina Grande. Prior to blood collection, the dog owners signed an informed consent form and answered a questionnaire in an interview conducted in order to identify possible risk factors associated with the disease. The research was approved by the Ethics Committee of Botucatu Medical School, UNESP (CEEA #897-2011).

Campina Grande is located on the semiarid region of Paraíba state, 125 km (77.67 miles) away from the capital, João Pessoa, and it has an area of 599.6 km^2^ (372.58 miles^2^) and average altitude of 555 m (1,820.87 ft) above the sea level. The population size was calculated by Epi Info 3.5.1 software, with 5 % of significance level (α), 95 % of confidence interval, and 6 % of margin of error [6]. The canine population of 55,000 animals was based on the human population of 385,000 inhabitants reported in the last census of the Brazilian Institute of Geography and Statistics (IBGE), in 2010 [7].

At the same time the blood was collected, a specific epidemiological questionnaire was filled out by their owners. *Leishmania* spp. antibodies were detected by the indirect fluorescent antibody test (IFAT), using *Leishmania major* and *Leishmania chagasi* antigens adopting 40 as the cutoff titer [8]. ELISA S7® recombinant kit, authorized by the Brazilian Ministry of Agriculture, Livestock and Food Supply (MAPA), was also employed, considering 100 UI as the cutoff titer.

Polymerase chain reaction (PCR) was performed using species-specific primers for *Leishmania* (*Leishmania*) *chagasi* (LC14: 5′-CGCACGTTATATCTACAGGTTGAG-3′; LC15: 5′-TGTTTGGGATTGAGGTAATAGTGA-3′), amplifying a final product of 167 base pairs (bp) from a preserved region of *Leishmania* (*Leishmania*) *chagasi* kDNA minicircle (FMVZ-UNESP). The PCR protocol and cycling was described by Lachaud et al. [9].

All data were tabulated in an Excel spreadsheet. The association of the results of *Leishmania* spp. antibodies and the epidemiological data was analyzed by chi-square (χ^2^) or Fischer’s exact test, adopting 5 % of significance level (α). The correlation between the antibody titers obtained by both tests was analyzed based on Spearman’s correlation coefficient (*rs*). Statistics related to the performance of IFAT-Lc, ELISA, and PCR were calculated by adopting IFAT-Lm as the gold standard [10]. All the analyses were performed by employing Epi Info 3.5.1 and BioEstat 5.0 software [11].

## Results and discussion

Thirty-three (8.4 %) dogs resulted positive to IFAT and 17 (4.3 %) to ELISA. Although the disease maintains its rural characteristics, nowadays its urbanization can be noticed.

A comparison of results obtained by IFAT with *Leishmania major* antigen presented better results than IFAT with *Leishmania chagasi* antigen with 98 % of efficiency, followed by ELISA, with 93 %. The results obtained by polymerase chain reaction showed that only eight (2 %) animals had *Leishmania* spp. DNA. CLV is a chronic disease and this result may be expected.

The highest prevalence of reactive animals to *L. major* antibodies was found in the neighborhoods of Palmeira, Dinamérica and Bodocongó, located in the suburbs, presenting areas with social and economic problems. From the seropositive animals, only one dog had clinical signs consistent with the disease characterized by alopecia, splenomegaly, malnutrition, conjunctivitis, and corneal opacity.

In the analysis of the studied epidemiological variables, only contact with other animals presented statistical association (*p* = 0.02; Table 1), demonstrating that dogs in contact with other animals are 4.1 times more likely to be seropositive to anti-*L. major* antibodies than animals that have no contact. When the results are stratified by diversifying the number of dogs without contact with any other animal, the prevalence was 1.7 %. In case of contact with only one animal, the prevalence was 6.3 and 15.2 % when there was contact with two or more animals (*p* <0.05). These results corroborate those of Amóra et al. [12] who also noted that the presence of other dogs facilitates the maintenance of infection among this species, which increases the risk of contamination to humans [13].

**Table 1:** Association between IFT-Lm results and epidemiological variables investigated in the studied animals

Variable		N	IFAT^a^	Variable (%); CI95%^b^	OR (CI95%)^c^	*p* ^d^
Gender	Male	195	14	7.2; 4.4–11.7	1.4 (0.6–2.9)	0.24^e^
	Female	196	19	9.7; 6.3–14.7		
Breed	Pure	114	9	7.9; 4.2–14.3	1.1 (0.5–2.5)	0.49^e^
	Mongrel	277	24	8.7; 5.9–12.6		
Age (years)	Young (≤1 year)	54	4	7.4; 3.0–17.6	1.2 (0.4–3.5)	0.51^e^
	Adult (>1 year)	337	29	8.6; 6.1–12.1		
Age (months)	6-12 months	54	4	7.4; 3.0–17.6	–	0.78^f^
	12-24 months	81	5	6.2; 2.7–13.7		
	24-48 months	92	7	7.6; 3.8–14.9		
	48-60 months	86	8	9.3; 4.8–17.3		
	>60 months	78	9	11.5; 6.2–20.5		
Contact with other animals	No	77	2	2.6; 0.8–9.0	4.1 (1.0–17.6)	0.02^e^
	Yes	314	31	9.9; 7.1–13.7		
Number of contacted animals	None	59	1	1.7; 0.4–8.9	–	0.00^f^
	Only one	207	13	6.3; 3.7–10.5		
	Two or more	125	19	15.2; 10.0–22.5		
Raising	Domestic	307	23	7.5; 5.1–11.0	–	0.27^f^
	Semi-domestic	79	10	12.7; 7.1–21.8		
	Free	5	0	0.0; 0.0–0.0		
Food	Commercial feed	88	8	9.1; 4.7–16.9	–	0.84^f^
	Homemade food	130	12	9.2; 5.4–15.5		
	Both	173	13	7.5; 4.5–12.4		
Environment	Earth	80	7	8.8; 4.4–17.0	–	0.91^f^
	Cement	178	16	9.0; 5.6–14.1		
	Both	131	10	7.6; 4.2–13.5		
Disinfection	Daily	326	25	7.7; 5.3–11.1	–	0.08^f^
	Weekly	46	8	17.4; 9.1–30.8		
	Fortnightly	15	0	0.0; 0.0–0.0		
	Monthly	3	0	0.0; 0.0–0.0		

^a^Titer ≥40; ^b^frequency of positive animals based on the variables studied (confidence interval = 95 %); ^c^OR: odds ratio; ^d^ *p*: *p* value for α = 5; ^e^Fisher’s exact test; ^f^ chi-square test

There was no statistical association (*p* >0.05) regarding dogs living in environments with daily, weekly, biweekly or monthly cleaning. It is known that cleaning at regular intervals prevents the accumulation of organic matter, which avoids the proliferation of phlebotomines that need organic matter to complete their life cycle (Table 1).

Regarding gender, females had a higher prevalence of 9.7 %, compared with males that had 7.2 %. However, there was no statistical association (*p* = 0.24), confirming the results of Barbosa et al. [14], who found higher prevalence in females, but without statistical association. The chances of infection are the same for both genders (Table 1).

Assessing the results regarding animals’ age and breed, there is no statistical association according to Barbosa et al. [14] and Amóra et al. [12]. However, Silva et al. [15] and Dantas-Torres et al. [5] found higher prevalence in young dogs (*p* <0.05). There was also no statistical association concerning breed, and then the prevalence was similar in all the situations (Table 1).

Statistical associations were not observed (*p* >0.05) in relation to the environment, the fact that the animal remained on dirt floor, cement or both; if commercial feed, homemade food or both were provided to the dog (Table 1); and regarding the education and income of the ones responsible for the animals (Table 2).

**Table 2:** Association between the social condition of the owners (education and income) and the detection of antibodies against *Leishmania major* by IFAT

Variable		N	IFAT^a^	Variable (%); CI95%^b^	OR (CI95%)^c^	*P* ^d^
Education	Illiterate	20	1	5.0; 1.2–23.8	–	0.76^f^
	Incomplete primary education	62	8	12.9; 6.7–23.5		
	Primary education	46	2	4.3; 1.3–14.5		
	Incomplete secondary education	75	7	9.3; 4.7–18.1		
	Complete secondary education	102	8	7.8; 4.1–14.7		
	Incomplete tertiary education	20	1	5.0; 1.2–23.8		
	Complete tertiary education	66	6	9.1; 4.3–18.5		
Income	<2 wages	197	20	10.2; 6.7–15.2	–	0.56^f^
	2–4 wages	130	10	7.7; 4.3–13.6		
	5–6 wages	38	2	5.3; 1.6–17.3		
	>6 wages	26	1	3.8; 0.9–19.0		

## Conclusion

CVL prevalence in Campina Grande has increased in the last years, showing that this disease has adapted to the urban area with an endemic profile, which shows the lack of knowledge by the population and the lack of attention regarding disease control. Animals that had contact with other animals are more likely to be serologically positive to *Leishmania major* antibodies.

### Ethics approval

The present study was approved by the Ethics Committee of Botucatu Medical School, UNESP, CEEA 897–2011.

## References

[1] Oliveira CI, Teixeira MJ, Gomes R, Barral A, Brodskyn C (2004). Animal models for infectious diseases caused by parasites: Leishmaniasis. Drug Discov Today: Dis Models.

[2] Harhay MO, Olliaro PL, Costa DL, Costa CH (2011). Urban parasitology: visceral leishmaniasis in Brazil. Trends Parasitol.

[3] González U, Pinart M, Sinclair D, Firooz A, Enk C, Vélez ID (2015). Vector and reservoir control for preventing leismaniasis. Cochrane Database Syst Rev.

[4] Pimentel DS, Ramos RA, Santana MA, Maia CS, de Carvalho GA, da Silva HP (2015). Prevalence of zoonotic visceral leishmaniasis in dogs in an endemic area of Brazil. Rev Soc Bras Med Trop.

[5] Dantas-Torres F, de Brito MEF, Brandão-Filho SP (2006). Seroepidemiological survey on canine leishmaniasis among dogs from an urban area of Brazil. Vet Parasitol.

[6] Epi ITM (2002). Atlanta: Centers for Diseases Control and Prevention.

[7] Demográfico C (2010). Brasil: Instituto Brasileiro de Geografia e Estatística.

[8] Camargo ME (1966). Fluorescent antibody test for the serodiagnosis of American trypanossomiasis: technical modification employing preserved culture forms of *Trypanossoma cruzi* in a slide test. Rev Inst Med Trop São Paulo.

[9] Lachaud L, Marchergui-Hammami S, Chabbert E, Dereure J, Dedet JP, Bastien P (2002). Comparison of six PCR methods using peripheral blood for detection of canine visceral leishmaniasis. J Clin Microbiol.

[10] Mackinnon A (2000). A spreadsheet for the calculation of comprehensive statistics for the assessment of diagnostic tests and inter-rater agreement. Comput Biol Med.

[11] Ayres M, Ayres JRM, Ayres DL, Santos AS (2007). BioEstat 5.0. Aplicações estatísticas nas áreas das ciências biológicas e médicas.

[12] Amóra SSA, Santos MJP, Alves ND, Costa SCG, Calabrese KS, Monteiro AJ (2006). Fatores relacionados com a positividade de cães para leishmaniose visceral em área endêmica do Estado do Rio Grande do Norte, Brasil. Cienc Rural.

[13] Gontijo CMF, Melo MN (2004). Leishmaniose Visceral no Brasil: quadro atual, desafios e perspectivas. Rev Bras Epidemiol.

[14] Barbosa DS, Rocha AL, Santana AL, Sousa CSF, Dias RA, Costa-Junior LM (2010). Soroprevalência e variáveis epidemiológicas associadas à leishmaniose visceral canina em área endêmica no município de São Luís, Maranhão, Brasil. Ciênc Anim Bras.

[15] Silva AVM, de Paula AA, Cabrera MAA, Carreira JCA (2005). Leishmaniose em cães domésticos: aspectos epidemiológicos. Cad Saúde Publ.

